# Editorial: Physiological telemonitoring and interventional telemedicine in extreme environments

**DOI:** 10.3389/fphys.2023.1353731

**Published:** 2024-01-05

**Authors:** C. Balestra, G. Bosco, D. Cialoni, J. Kot, R. Pelliccia, A. Marroni

**Affiliations:** ^1^ DAN Europe Research Division, Brussels, Italy; ^2^ Environmental, Occupational, Aging (Integrative) Physiology Laboratory, Haute Ecole Bruxelles-Brabant (HE2B), Brussels, Belgium; ^3^ Motor Sciences Department, Physical Activity Teaching Unit, Université Libre de Bruxelles (ULB), Brussels, Belgium; ^4^ Environmental Physiology and Medicine Lab, Department of Biomedical Sciences, University of Padova, Padua, Italy; ^5^ National Centre for Hyperbaric Medicine Institute of Maritime and Tropical Medicine, Medical University of Gdansk, Gdynia, Poland

**Keywords:** real-time physiological monitoring, diving, telemedicine, DCS prevention, extreme environments

## Introduction

Telemetric systems, including wearable, implantable, and consumable sensors, allow extensive health-related monitoring not yet available in all conditions to which humans are exposed.

An important goal in the sustainability of the individual’s proper health and performance in both dry and wet extreme environments is the ability to directly access key physiological and vital data, such as heart and breathing rates, body temperature, and stress-related components in blood, urine, and saliva, in real time.

During breath-hold diving (BHD), for example, the arterial partial pressure of oxygen and carbon dioxide is believed to progressively increase during descent, as explained by theory and previous end-tidal alveolar gas measurements and arterial blood gas (ABG) analysis in hyperbaric chambers. Recent ABG experiments in real underwater environments found a paradoxical drop at depth in some divers. This confirms that some BHD divers can experience hypoxemia at depth. The hypothesized explanation for such a discrepancy is lung atelectasis, as suggested by lung ultrasound findings performed at depth.

Divers encounter inert gas narcosis (IGN) at depth, exhibiting a marked loss in circulating dopamine levels, likely accounting for brain-derived neurotrophic factor (BDNF)-dependent impairment of mental capacity and heightened oxidative stress indicators in blood and saliva. The decline in dopamine and BDNF levels appears to persist at decompression under dry conditions; thus, boosting dopamine/BDNF signaling via pharmacological or other intervention types might attenuate IGN in deep dives (Balestra et al., 2012; Lafere et al., 2016; Rocco et al., 2019; Bosco et al., 2023).

Saturation diving allows divers to reduce the risk of decompression sickness, while working at depth for prolonged periods, but may increase reactive oxygen species (ROS) production. Such modifications can affect endothelial function by exacerbating oxidative stress and have been measured from saliva and urine samples (Mrakic-Sposta et al., 2020; Imbert et al., 2022).

## Observations and recent results

As adventurous individuals explore extreme environments, they are faced with expected and unexpected challenges. Challenging environmental constraints can also arise in normal daily life as a result of climatic change, progressive aging effects, viral pandemics, and even natural disasters, and can trigger similar adaptive and coping mechanisms.

**FIGURE 1:**
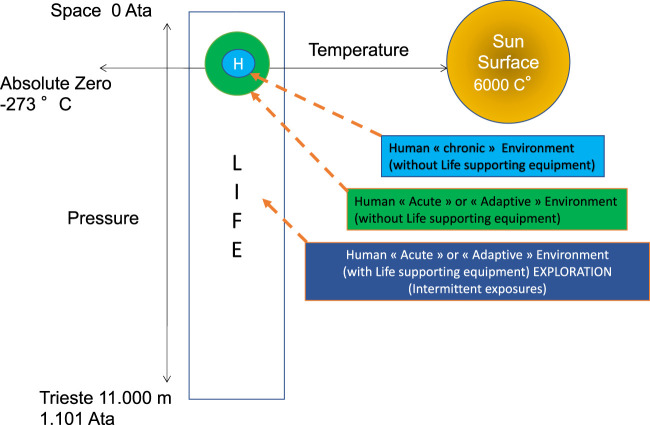
Human exploratory possibilities in “Chronic” or “Acute” environments (reproduced from Balestra et al. (2018) under Creative Commons license).

Humans inhabit a confined “chronic” environment to which they are naturally adapted (see Figure 1) and can support acute, short-term excursions beyond their “usual” environment until life support systems are required. For example, environmental conditions and other factors can adversely affect oxygen availability and, consequently, cause variations that drive the oxygen concentration far from its tight physiological “optimum.” Independent of the total amount of oxygen that reaches specific tissues to fulfill designated cell functions, the optimal oxygen concentration seems to be restricted to a narrow range for each cell type.

The average human settlements are found at or near sea level (∼20.8% oxygen concentration). Despite the fall in atmospheric pressure (and inspired oxygen pressure) with increased altitude above sea level, human settlements are sustainable up to 2,400–2,700 m above sea level; here, the inhabitants live in a chronic state of hypoxia (∼15% effective oxygen). An extreme example of a permanent human habitation on the edge of sustainability is the gold mining town of La Rinconada in Peru centered at ∼5,000 m above sea level. The low atmospheric pressure restricts the residents to only approximately 11% of oxygen in breathable air at sea level.

For many years, diving and hyperbaric medicine have strived to increase our understanding of the effects of environmental stressors on human pathophysiology. This effort has demonstrated the importance of the very reactive oxygen element, named oxy-gene or “acid generator” by the Greeks, and the chemically bound and life-promoting oxygen molecule (O_2_). Diving is an activity that places humans in a “challenging environment” in which specific life-maintaining systems are required for temporary exposures (see Figure 1). It is probably the only common recreational, military, or commercial activity (outside of the medical field) that will expose a considerable number of people for a non-negligible time to oxygen levels higher than atmospheric levels (Balestra et al., 2023).

Breathing air at hyperbaric pressure can lead to oxidative stress with generation of reactive oxygen species (ROS) and reactive nitrogen species (RNS). Oxidative stress has been investigated in both breath-hold diving (Cialoni et al., 2021) and scuba diving (Cialoni et al., 2019), with particular regard to the dive depth phase.

It is clear that measuring vital signs or any other useful data that can be telemonitored is essential during our stay in challenging environments.

Heart rate variability (HRV) during underwater diving has been infrequently investigated largely due to environmental limitations and technical challenges. A recent study presented in this special topic (Lafere et al., 2016) aims at analyzing HRV changes while diving at variable hyperoxic levels when using an open-circuit (OC) air diving apparatus or at constant hyperoxia using a closed-circuit rebreather (CCR).

This Research Topic addresses several articles focusing on human heart rate variability (HRV) as the vital parameter that reflects both sympathetic (SNS) and parasympathetic neural system (PNS) activation and also the interaction between cardiovascular and respiratory activities. Measurement of HRV while underwater demonstrates its feasibility, accuracy, and interest.

Lafère et al. showed an interesting approach of non-linear analysis of HRV including fractal dimension changes. This non-linear approach permits to reduce the incidence of sinus respiratory arrhythmia which is clearly enhanced during underwater breathing due to increased work of breathing and augmented dead space that pushed to have greater respiratory amplitude. The authors showed a possibility to link HRV fractal dimension changes to oxygen partial pressure variations.

In the same direction, Lundell et al. discussed the role of different parts of the diving reflex in contributing to parasympathetic nervous system activation while diving with a closed-circuit rebreather under very cold conditions. Their findings indicate a biphasic answer: the trigeminocardiac part of the diving reflex causes the strong initial PNS activation at the beginning of the dive, but the reaction seems to decrease quickly. After this initial activation, cold seemed to be the most prominent promoter of PNS activity—not pressure.

Having observed a concurrent increase in both SNS and PNS branches, the authors associated these changes with an elevated risk for arrhythmia during effort and recommended a short adaptation phase at the beginning of cold water exposure and before physical activity.

Acute hypoxia during breath-hold diving is potentially life-threatening and must be avoided, say when athletes want to push their limits during competitions. Mulder et al. developed an elegant safety device to mitigate the risk of hypoxic blackout (BO) during the last part of the ascent from apnea dive. They investigated pulse oxygen saturation (SpO_2_) and heart rate (HR) in shallow and deep free dives. All divers desaturated more during deeper dives (nadir 55% ± 10%) compared to shallow dives (nadir 80% ± 22%) with a lowest SpO_2_ of 44% in one deep dive. HR showed a “diving response,” with a similar lowest HR of 42 bpm in shallow and deep dives; the lowest value (28 bpm) was observed in one shallow dive. They convincingly showed that continuous pulse oximetry monitoring as part of essential variables collected underwater may be an important step to increase free-diving safety.

Newly published data using more invasive methods by Paganini et al. (2023) provide additional understanding of the often disregarded complex physiological changes and adaptation phenomena to dysbaric exposure.

Lastly, it is always essential to integrate and apply established and new basic science and clinical findings to advance the understanding of such an important field of medical research. Paganini et al. appropriately addressed the importance of understanding through the use of simulation in specific environments. They proposed realistic scenarios and appropriate scientific references that will be of interest for more knowledge building and intervention’s efficiency.

## Conclusion

The application of modern sensing technologies opens new frontiers in the expansion of human presence in ever more extreme environments. This Research Topic explores the challenges facing humans during underwater activities, and the articles offer a collective snapshot of the advances being made to provide continuous, non-invasive acquisition of vital physiological measurements in real time.
